# Case Report: Cardiac Toxicity Associated With Immune Checkpoint Inhibitors

**DOI:** 10.3389/fcvm.2021.727445

**Published:** 2021-12-06

**Authors:** Ru Chen, Ling Peng, Zhihua Qiu, Yan Wang, Fen Wei, Min Zhou, Feng Zhu

**Affiliations:** ^1^Department of Cardiology, Union Hospital, Tongji Medical College, Huazhong University of Science and Technology, Wuhan, China; ^2^Clinic Center of Human Gene Research, Union Hospital, Tongji Medical College, Huazhong University of Science and Technology, Wuhan, China; ^3^Cancer Center, Union Hospital, Tongji Medical College, Huazhong University of Science and Technology, Wuhan, China; ^4^Department of Pulmonary and Critical Care Medicine, Tongji Hospital, Tongji Medical College, Huazhong University of Science and Technology, Wuhan, China; ^5^Key Laboratory of Respiratory Diseases, National Ministry of Health of the People's Republic of China and National Clinical Research Center for Respiratory Disease, Wuhan, China

**Keywords:** immune checkpoint inhibitors, cancer, myocardial injury, immune-related adverse events, myocarditis

## Abstract

Immune checkpoint inhibitors (ICIs) have now emerged as a mainstay of treatment for various cancer. Along with the development of ICIs, immune-related adverse effects (irAEs) have been the subject of wide attention. The cardiac irAE, a rare but potentially fatal and fulminant effect, have been reported recently. This article retrospectively reviewed 10 cases from our hospital with cardiac irAEs, with severity ranging from asymptomatic troponin-I elevations to cardiac conduction abnormalities and even fulminant myocarditis. In our series, all the cases were solid tumors and lung cancer was the most frequent cancer type (4,40%). In total, three (30.0%) patients experienced more than one type of life-threatening complication. A systemic corticosteroid was given to nine patients (90.0%). The majority of cases (7, 70%) were performed at an initial dose of 1–2 mg/kg/day. Two (20.0%) patients were admitted to ICU, three (30.0%) patients were put on mechanical ventilation, two (20.0%) patients received the plasma exchange therapy, and one patient was implanted with a pacemaker. Two (20.0%) of the patients succumbed and died, with a median duration of 7.5 days (IQR5.0–10.0) from diagnosis of cardiac irAE to death. Based on these results, we recommend that clinicians be alert to cardiac irAEs, including performing cardiovascular examinations before ICI treatment to accurately diagnose suspected myocarditis, enabling immediate initiation of immunosuppressive therapy to improve prognosis.

## Introduction

Immune checkpoints are involved in the key negative regulatory pathways of immune surveillance, aiding tumor cells to avoid immune detection and destruction. Immune checkpoint inhibitors (ICIs) are monoclonal antibodies targeting blocked immune checkpoint proteins such as cytotoxic T lymphocyte-associated antigen 4 (CTLA-4), programmed cell death 1 (PD-1), and PD-1 ligand (PD-L1) (1). ICIs increase T cell activation as well as T-cell mediated anti-tumor effect, and have become a revolutionary treatment that prolongs the survival of various malignancies previously endowed with poor prognosis over the last decade (2). Along with the prosperity of ICIs, system-wide immune-related adverse events (irAEs) have emerged and drawn increasing attention. Dermatologic, hepatic, gastrointestinal, and endocrine toxicities are among the most common irAEs reported in patients treated with monotherapy or combined ICIs therapy (3).

Cardiotoxicity, one of the rare irAEs, used to be underestimated and has roused clinicians' attention in recent years. The incidence of ICIs related myocarditis varies from 0.1 to 1% (4, 5). However, the mortality rate of ICI-related myocarditis ranges from 35 to 50% (6, 7). Over the past few years, several cases of myocarditis associated with ICIs have been reported, and most of the patients presented unspecific manifestations, along with atypical laboratory abnormalities and imaging features (8). Despite treatment with high doses of steroids, the poor outcome was fatal in some cases. Because it is rare but potentially fatal with a fulminant effect, it is important to characterize cardiac irAEs in an early stage. This study presents a series of 10 cases that developed ICI related cardiac irAEs, as well as subsequent treatment and outcomes.

## Materials and Methods

A retrospective case study was performed on patients who presented with ICI related myocardial injury at Union Hospital in the Tongji Medical College of Huazhong University of Science and Technology, Wuhan (China) from January 1, 2018, to August 30, 2021. ICI related myocarditis was defined according to the criteria proposed by Marc P. Bonaca et al. (9) and classified as definite myocarditis, probable myocarditis, and possible myocarditis.

The study was approved by the Ethics committee of the Tongji Medical College of Huazhong University of Science and Technology (China). Data were retrieved from electronic medical records including demographic features, clinical features, laboratory findings, and images. Two physicians (Zhihua Qiu and Fen Wei) independently reviewed the data.

## Results

### Demographic and Clinical Characteristics of Enrolled Cases With Cardiac irAE

This study enrolled 10 patients with ICI-related cardiac cardiotoxicity who were admitted to the hospital between January 1, 2018, and August 30, 2021. The detailed descriptions of each case are provided in the Supplementary Materials, and the characteristics are displayed in Supplementary Table 1.

As shown in Table 1, the median age of patients was 62.5 (55.0–73.0) years old. Half of the cases were male. Four (40%) patients were diagnosed with lung cancer and 2 patients were diagnosed with thymoma. Other cancer types included cervical cancer, pyriform sinus squamous cell carcinoma, gallbladder carcinoma, and hepatocellular carcinoma. Furthermore, one patient diagnosed with cervical cancer had a previous history of breast cancer, and one patient with lung adenocarcinoma was once diagnosed with papillary thyroid carcinoma. Before ICI treatment, 70% of the patients were treated with surgeries, and 80% had received chemotherapy. Radiotherapy was performed on 60% of the patients, and two (20%) patients also received angiogenesis inhibitors. Only two patients were found to have previous cardiovascular disease, one was diagnosed with hypertension and the other was diagnosed with coronary heart disease.

**Table 1:** Demographic and base line clinical characteristics of the patients with cardiac irAE.

**Characteristic**	**Patients (*n*=), Median (IQR), No. (%)**
**Median age (IQR), year**	62.5 (55.0–73.0)
**Male sex**	5 (50.0)
**Tumor diagnosis**	
Lung cancer	4 (40.0)
Thymoma	2 (20.0)
Cervical cancer	1 (10.0)
Pyriformsinus squamous cell carcinoma	1 (10.0)
Gallbladder carcinoma	1 (10.0)
Hepatocellular carcinoma	1 (10.0)
**History of prior treatment**	
Operation	7 (70.0)
Chemotherapy	8 (80.0)
Radiotherapy	6 (60.0)
Angiogenesis inhibitors	2 (20.0)
Interventional therapy	2 (20.0)
**Co-morbidities**	
Hypertension	1 (10.0)
Coronary heart disease	1 (10.0)
**Types of ICI**	
**Anti-PD-1 antibody**	
Sintilimab	3 (30.0)
Tislelizumab	1 (10.0)
Camrelizumab	3 (30.0)
Pembrolizumab	2 (20.0)
**Anti-PD-L1 antibody**	
ZKAB001	1 (10.0)
**Days from first dose of ICI to onset**	28.5 (17.8–63.5)
**Symptoms and signs on admission**	
Fever	1 (10.0)
Palpitation	3 (30.0)
Chest tightness	5 (50.0)
Dyspnea	2 (20.0)
Fatigue	3 (30.0)
**Neuromuscular symptom**	
Blepharoptosis	4 (40.0)
Diplopia	1 (10.0)
Conscious disturbance	1 (10.0)
**Types of cardiac irAE**	
Definite myocarditis	2 (20.0)
Probable myocarditis	0 (0.0)
Possible myocarditis	2 (20.0)
other myocardial injury	6 (60.0)
**Grade of cardiac irAE**	
G1-2	6 (60.0)
G3-4	2 (20.0)
G5	2 (20.0)

*IQR, interquartile range; CHD, coronary heart disease; ICI, immune checkpoint inhibitor; irAE, immune-related adverse event*.

The most frequent ICIs treatment for the cases were sintilimab (30%) and camrelizumab (30%). Nine patients were treated with anti-PD-1 therapy, while the remaining one received anti-PD-L1 therapy. Median time from the use of the first dose of ICI to onset was 28.5 (17.8–63.5) days and symptoms at onset varied markedly. The most common symptoms presented in our cases were chest tightness, which occurred in 50% of the patients. Palpitations and fatigue were presented in 30% of the patients. Neuromuscular symptoms also occurred in ICI-related cardiac irAE. Forty percentage of the patients presented with blepharoptosis, one patient showed diplopia and one patient developed conscious disturbance during the disease.

Based on the diagnosing criteria proposed by Marc P. Bonaca et al. (9), only two patients were classified as having definite myocarditis. Six cases were classified as other myocardial injury and two patients were regarded as having possible myocarditis, mainly due to the lack of confirmed examinations such as coronary angiography to rule out acute coronary syndrome. Sixty percentage of the patients were mild cases classified as G1-2.

### Laboratory, Electrocardiogram, and Echocardiogram Findings of Enrolled Cases With Cardiac irAE

Laboratory, electrocardiogram, and echocardiogram findings for the patients with cardiac irAE were shown in Table 2. Lymphopenia was frequently found in the cardiac irAE (7, 70%). The elevated level of serum alanine aminotransferase (ALT) and aspartate aminotransferase (AST) was observed in six (60%) and nine (90%) patients, respectively. Markedly evaluated levels of creatinine kinase (CK), creatinine kinase-muscle and brain type (CK-MB), and troponin I (TnI) were found in nine (90%) patients. B-type natriuretic peptide (BNP) tests were available in eight patients, and levels were significantly elevated in four (50%) of them. The serum levels of C-reactive protein (CRP), an important biomarker in systemic inflammation, were available in five patients, and CRP levels were elevated in four (80%). Four heart-specific autoantibodies were measured in two patients (Case 1 and 4, see Supplementary Material 1) by enzyme-linked immunosorbent assay, and both of their serum samples were positive.

**Table 2:** Laboratory tests and echocardiogram findings of the patients with cardiac irAE.

**Last lab test indexes before Steroids (*U*; normal range), change**	**Median (IQR), No. (%)**
Leucocytes (× 10^9^ per L; 3.5–9.5), Increased	7.4 (5.6–9.2), 3 (30.0)
Lymphocytes (× 10^9^ per L; 1.1–3.2), Decreased	1.0 (0.7–1.1), 7 (70.0)
Neutrophils (× 10^9^ per L; 1.8–6.3), Increased	5.8 (3.7–7.1), 5 (50.0)
**Lab test indexes at peak (** ***U*** **; normal range)**	
ALT (U/L; 9.0–50.0), Increased	135.0 (32.5–212.3), 6 (60.0)
AST (U/L; 15.0–40.0), Increased	168.0 (49.8–544.8), 9 (90.0)
LDH (U/L; 120.0–250.0), Increased	765.0 (414.5–1223.8), 9 (90.0)
Hs-TnI (ng/L; <26.2), Increased	1,252.1 (213.7–20058.5), 9 (90.0)
CK-MB (ng/mL; <6.6), Increased	75.0 (10.9–417.7), 9 (90.0)
CK (U/L; 38–174), Increased	3,573.0 (662.3–9,900.0), 9 (90.0)
BNP (pg/ml, <100), Increased	178.9 (25.3–369.8), 4 (50.0), *N* = 8
C-reactive protein (mg/L; 0.0–10.0), Increased	42.7 (9.8–82.4), 4 (80.0), *N* = 5
Positive autoantibodies against β1-AR	2 (100.0), *N* = 2
Positive autoantibodies against L-type calcium channel	2 (100.0), *N* = 2
Positive autoantibodies against MHC	2 (100.0), *N* = 2
Positive autoantibodies against ANT	2 (100.0), *N* = 2
**Electrocardiogram findings**	**No. (%)**
Sinus tachycardia	8 (80.0)
Atrial extrasystole	2 (20.0)
Ventricular extrasystole	0 (0.0)
Atrioventricular block	4 (40.0)
Bundle branch block	3 (30.0)
ST segment elevation	2 (20.0)
Others^a^	4 (40.0)
**Echocardiogram findings**	**No. (%)**
Reduced ejection fraction	3 (30.0)
Cardiac dilatation	2 (20.0)
Wall motion abnormality	3 (30.0)

*ALT, Alanine aminotransferase; AST, Aspartate aminotransferase; LDH, Lactate dehydrogenase; Hs-TnI, high-sensitivity troponin I; CK-MB, creatine kinase-muscle and brain; CK, creatine kinase; BNP, type B natriuretic peptide*.

a *Indicates unspecific ST-T change and Q wave*.

12-lead ECG and transthoracic echocardiography were performed in all cases. Cardiac conduction disorders including atrioventricular block (AVB) and bundle branch block were found in seven (70%) patients. One patient presented I-II°AVB, which eventually progressed to complete AVB (case 1, Supplementary Material 1). Systolic function was preserved in seven (70%) patients, and only three (30%) patients developed systolic dysfunction with left ventricular hypokinesia.

### Treatment and Clinical Outcome of the Patients With Cardiac irAE

As shown in Table 3, systemic corticosteroid was given to nine patients (90.0%). Methylprednisolone was initiated on the 9th day (IQR 7.0–15.0) after symptom onset or detection of increased serum markers. The majority of cases (6, 60%) were treated at an initial high dose of 1–2 mg/kg/day. Two cases (case 3 and 4) initiated 500–1,000 mg daily methylprednisolone because of multiple life-threatening complications, and another case (case 9) was prescribed initial methylprednisolone at 0.5 mg/kg/day due to no symptom and mild elevation of CK-MB. The median duration of corticosteroids in our series was 5.0 weeks (IQR 5.0–6.0). Moreover, intravenous immunoglobin was prescribed to two patients (10%). Therapy for heart disease included ACEI (1, 10%), β-blocker (5, 50%), trimetazidine (6, 60%), and coenzyme Q10 (4, 40%).

**Table 3:** Treatment and clinical outcome of the patients with cardiac irAE.

**Treatment**	**No. (%), median (IQR)**
**Medicine therapy**	
Systemic Steroids	9 (90.0)
Immunoglobin	2 (20.0)
AECI/ARB/ARNI	1 (10.0)
β blockers	5 (50.0)
Trimetazidine	6 (60.0)
Coenzyme Q10	4 (40.0)
**Advanced therapy**	
Mechanical ventilation	3 (30.0)
Plasma exchange	2 (20.0)
Pacemaker	1 (10.0)
ECMO	0 (0.0)
**Admission to ICU**	2 (20.0)
**Complications**	5 (50.0)
Single type (myasthenia)	1
Multiple types (CCD, heart failure, respiratory failure, cardiac arrest, and myasthenia)	
2^a^ types	2 (20.0)
4^b^ types	1 (10.0)
5^c^ types	1 (10.0)
**Clinical symptoms evaluation**	
Improvement	8 (80.0)
Worse	2 (20.0)
**Clinical outcomes**	
Discharge from hospital	8 (80.0)
Death	2 (20.0)
Hospital stay (Pts. discharge), days	10.5 (5.75–23.0)
Time from diagnosis of cardiac irAE to death, days	7.5 (5.0–10.0), *N* = 2
**Rechallenge ICIs**	
Yes	1 (10.0)
No	7 (70.0)

*IQR, interquartile range; AECI/ARB/ARNI, angiotensin II converting enzyme inhibitors/angiotensin receptor blocker/Angiotensin receptor neprilysin inhibitor; ECMO, extracorporeal membrane oxygenation; ICU, intensive care unit; CCD, cardiac conduction defect; ICIs, immune checkpoint inhibitors*.

a *1 case with heart failure and myasthenia, the other case with respiratory failure, and myasthenia*.

b *1 case complicated with CCD, heart failure, respiratory failure, and cardiac arrest*.

c *1 case complicated with CCD, heart failure, respiratory failure, cardiac arrest, and myasthenia*.

As the disease progressed, four (40.0%) patients had life-threatening complications. Three patients experienced more than one type of complication, and one case experienced five types of complication including CCD with unstable hemodynamics, heart failure, respiratory failure, cardiac arrest, and myasthenia.

During the course two (20.0%) patients were admitted to ICU, three (30.0%) patients were put on mechanical ventilation, two (20.0%) patients received plasma exchange therapy, and one patient was implanted with a pacemaker. None of the cases received extracorporeal membrane oxygenation (ECMO) in our series.

Eight (80.0%) patients had been discharged with a median hospital stay of 10.5 days (IQR 5.75–23.0). However, two (20.0%) of the patients succumbed and died with a median time from diagnosis of cardiac irAE to death 7.5 days (IQR5.0–10.0). Only one patient was advised to rechallenge ICI therapy in the follow-up period.

## Discussion

The main cardiotoxicity associated with ICIs is myocarditis (10). The presentation of ICI related myocarditis encompasses a wide spectrum of symptoms including chest pain, chest distress, heart failure, and palpitations, etc. An elevation of cardiac biomarkers (troponin, CK, CK isoforms, and even BNP) was found in most patients (9). ECG changes were broad, including arrhythmia (atrial tachyarrhythmia, premature ventricular contractions, and ventricular tachycardia, ST-T wave abnormalities, PR segment changes, cardiac conduction defects, and so on) (11). Patients with ICI related myocarditis may present changes including left ventricular dysfunction and segmental wall motion abnormality in echocardiography however it is non-sensitive and unspecific (12). Cardiac magnetic resonance imaging (CMR) is preferred to echocardiography when diagnosing myocarditis. The presence of late gadolinium enhancement (LGE) was 48% in patients and the presence of elevated T2-weighted short tau inversion recovery (STIR) was 28% (13). Endomyocardial biopsy typically demonstrates infiltration of CD8+ T-cells, CD68+ macrophages, and signs of myocardial fibrosis (14).

By integrating all the related clinical characteristics, the uniform diagnostic criteria for ICI related myocarditis were proposed in 2019 (9). According to the principle, only two of our cases were definite myocarditis. Based on current criteria, evidence from cardiac biopsy, CMR and ultrasound are vital in diagnosing definite myocarditis. However, cardiac biopsy is hard to perform not only due to its technical difficulty but also the poor health condition of most cancer patients. CMR and ultrasound are easier to access, but in our cases, patients who underwent CMR showed no instructional abnormality and most echocardiograms showed no change compared to the previous results. Therefore, it is sometimes confusing in clinical work to give a definite diagnosis when confronted with myocardial injury after ICI treatment, especially when some rule out the need for potentially harmful examinations such as coronary CTA or angiography. Apart from myocarditis, another cardiotoxicity related to ICI has been reported. B.P. Geisler et al. reported a case of takotsubo-like syndrome in a melanoma patient treated with ipilimumab (15). ICI-induced hypertension, symptomatic sinus tachycardia, and angina pectoris have also been reported (16). In our cases, one patient developed I°AVB without elevation of hsTnI. Thus, a comprehensive understanding of ICI related cardiotoxicity and specific biomarkers for each cardiac irAEs are needed.

While T cell-mediated cardiac impairment is considered the overarching mechanism for ICI-related myocardial injuries, the PD-1 deficiency animal models suggest a potential role of heart-specific autoantibodies involved in myocardial injuries. Genetic deletion of PD-1 led to spontaneous dilated cardiomyopathy caused by autoantibodies targeting cardiac troponin I in BALB/c mice (17, 18). PD-1 deficiency in autoimmune Murphy Roths Large mice led to the development of fatal myocarditis with T cell and macrophage infiltration and generation of high-titer autoantibodies against cardiac myosin (19). There has also been an increasing number of reports regarding irAEs with the autoantibody profiles traditionally associated with autoimmune diseases, such as myositis (20), hypothyroidism (21), dermatitis (22, 23), and nephritis (24). However, to date, antibody-antigen deposits were not found in biopsy specimens from ICI-associated myocarditis in humans (8). Interestingly, we found two cases that were positive for circulating heart-specific autoantibodies related to heart failure. These autoantibodies can influence cardiac function by negative chronotropic and/or negative inotropic effects. Removing or neutralizing circulating autoantibodies by immunoadsorption or intravenous immunoglobulins may improve cardiac function and reduce morbidity in patients with heart failure (25). Further studies are warranted to elucidate the generation of circulating autoantibodies against heart and autoantibody-mediated damage on cardiac function in ICI-related myocardial injuries.

High dose steroids are currently regarded as the first-line treatment for rescuing the unspecific hyperactive immune response caused by ICI therapy. It was recommended that initiated 1–2 mg/kg/d of methylprednisolone followed by an oral steroid tapered slowly could be effective (26). However, it might be insufficient to deal with severe ICI-related cardiotoxicity by steroids alone. The other immunosuppressive therapies including plasmapheresis, intravenous immunoglobulins, mycophenolate, tacrolimus, and infliximab also should be considered if the patients get worse after steroid treatment for 24 h (27). Patients should also be treated with conventional therapy for each cardiovascular manifestation such as heart failure. In our cases, most patients responded well to steroid therapy, and traditional therapy for myocarditis including large doses of vitamin C, trimetazidine, and coenzyme Q10, might also help.

Compared with chemotherapy or radiotherapy, ICIs significantly improve the durable response rate and prolongs long-term survival with relatively limited adverse effects in both monotherapy and combination therapy for advanced cancers. In the last 3 years, ~11 kinds of ICIs have been approved for clinical application in various advanced cancers worldwide, as well as in China. More interdisciplinary physicians have also performed ICIs due to their accessibility and relatively low economic burden, especially for domestic ICIs drugs. In our study, we found that the systemic steroids were initiated 9 days (IQR 7.0–15.0) after symptom onset or detection of increased serum marker, which is not a relative early initiation time (6). This indicated relatively insufficient familiarity with adverse reactions for interdisciplinary physicians. The management of adverse events implicated assessment significantly, including cardiac baseline assessment, subsequent monitoring, multidisciplinary consultation, high clinical suspicion, and early diagnosis. Especially for ICI related myocarditis, which is a rare but potentially fatal and fulminant side effect, it is imperative to transfer patients in which this is suspected to a cardiology department or ICU for early improvement. Furthermore, currently, there is no treatment regime for different clinical types of ICI related myocardial injury that has been tested by randomized clinical trials. Perspective cohorts and randomized clinical trials are required to gain further information and standardize the management of ICI induced cardiac irAEs.

There are several limitations to our study. First, because four of our cases were asymptomatic and three of our cases were fulminant, it was challenging to perform confirmation tests (such as myocardial biopsy, CMR and coronary tests) in every case. Therefore, several cases could only be classified as unspecific myocardial injuries (9). Second, cases were collected from different sub-specialty departments and were of different severity, which restricted our overall analysis. Third, we collected cases mainly from 2018 to 2019, when guidelines were lacking for the diagnosis and treatment of ICI related myocardial injury.

To sum up, ICIs are currently increasingly utilized for a wide variety of malignancies. Along with the prosperity of ICIs, clinicians need to be aware of irAEs especially cardiac toxicities because of the potentially high risk for mortality in the short term.

## Data Availability Statement

The original contributions presented in the study are included in the article/Supplementary Material, further inquiries can be directed to the corresponding author/s.

## Ethics Statement

Written informed consent was obtained from the individual(s) for the publication of any potentially identifiable images or data included in this article.

## Author Contributions

RC and LP collected all the clinical data. ZQ and FW independently reviewed the data. RC and YW prepared the manuscript. MZ and FZ proposed the idea for this work. All authors contributed to the article and approved the submitted version.

## Funding

This work was supported by grants from the National Natural Science Foundation of China (No. 81570348).

## Conflict of Interest

The authors declare that the research was conducted in the absence of any commercial or financial relationships that could be construed as a potential conflict of interest.

## Publisher's Note

All claims expressed in this article are solely those of the authors and do not necessarily represent those of their affiliated organizations, or those of the publisher, the editors and the reviewers. Any product that may be evaluated in this article, or claim that may be made by its manufacturer, is not guaranteed or endorsed by the publisher.

## Supplementary Material

Supplementary Figure 1 Clinical characteristics of case 1. **(A)** Typical electrocardiograms **(ECG)** of case 1: ECG on the left indicated II° atrioventricular block; ECG on the right side indicated III° atrioventricular block. **(B)** SPECT of case 1 showed extensive myocardial perfusion reduction. **(C)** Serum level of hsTnI and CK-MB. **(D)** Endomyocardial biopsy of the patient showed cardiomyocyte degeneration and mild effusion.

Supplementary Figure 2 Clinical characteristics of case 2. **(A)** Serum level of hsTnI and CK-MB. **(B)** Typical ECGs of case 2 showed sinus tachycardia on the left side and III°AVB and elevation of ST segment in I and aVL on the right side.

Supplementary Figure 3 Clinical characteristics of case 3. **(A)** Typical electrocardiogram of case 3 showed elevated ST segment in II, III, aVF and V1-6 leads and complete right bundle branch block. **(B)** Serum level of hsTnI and CK-MB. **(C)** Typical electrocardiogram of case 3 showed complete right bundle branch block.
