# Supplementary figures and images for: Case Report: Cardiac Toxicity Associated With Immune Checkpoint Inhibitors
Source: Front Cardiovasc Med. 2021 Dec 6;8:727445. doi: 10.3389/fcvm.2021.727445 (PMC8685438; doi:10.3389/fcvm.2021.727445)

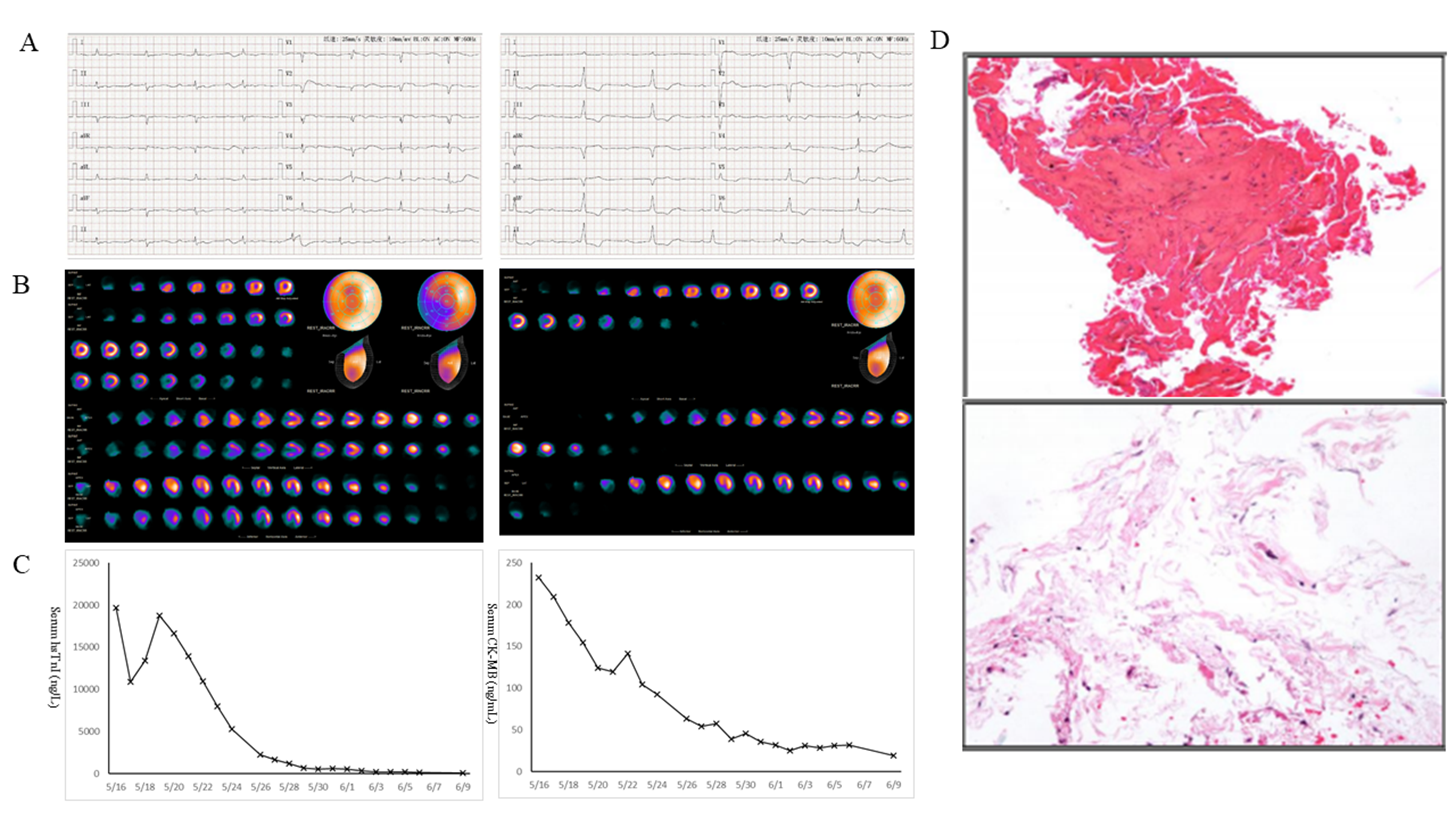

Supplement: Supplementary Figure 1 — Clinical characteristics of case 1. (A) Typical electrocardiograms (ECG) of case 1: ECG on the left indicated II° atrioventricular block; ECG on the right side indicated III° atrioventricular block. (B) SPECT of case 1 showed extensive myocardial perfusion reduction. (C) Serum level of hsTnI and CK-MB. (D) Endomyocardial biopsy of the patient showed cardiomyocyte degeneration and mild effusion. [file Image_1.TIF]

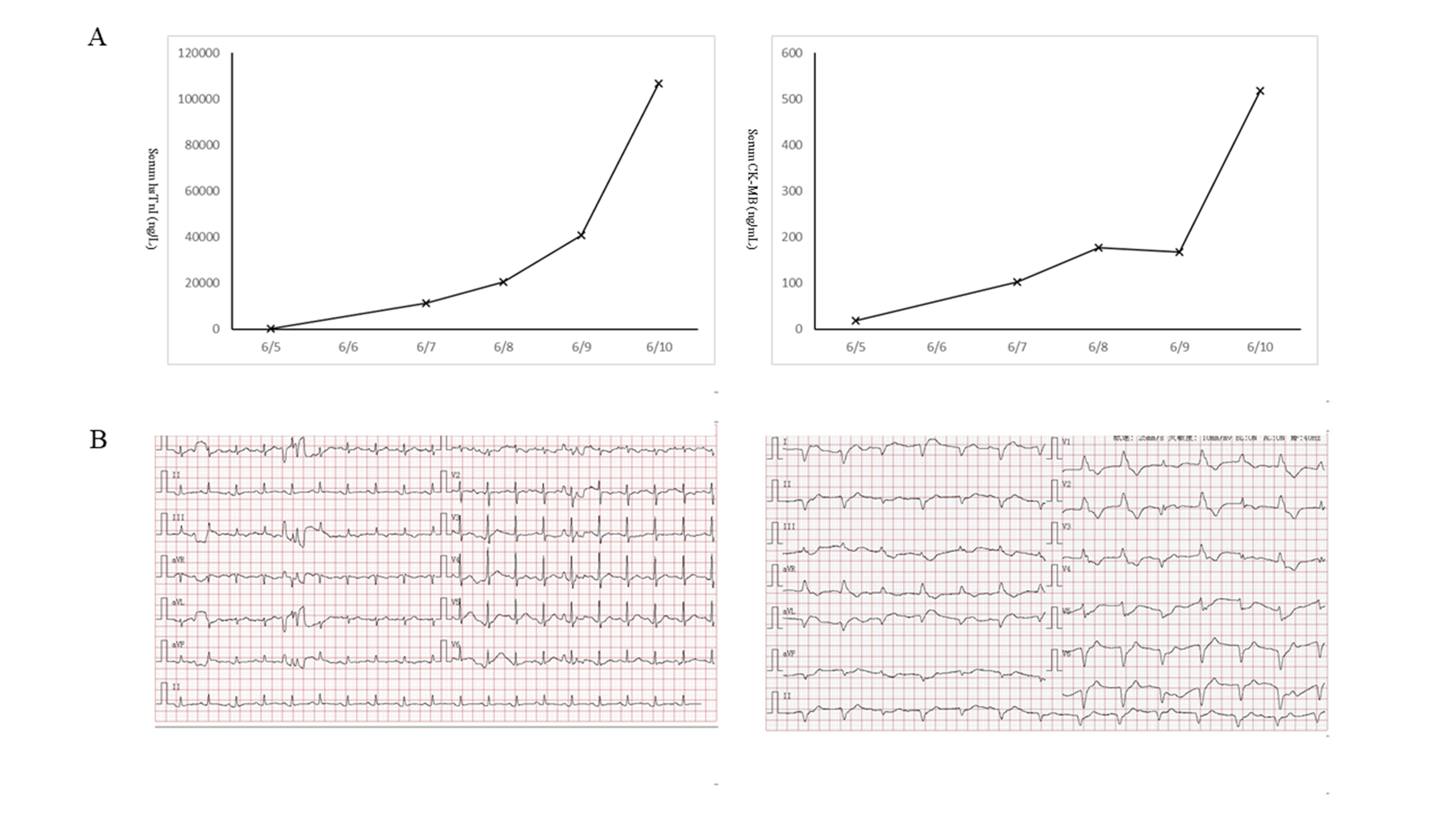

Supplement: Supplementary Figure 2 — Clinical characteristics of case 2. (A) Serum level of hsTnI and CK-MB. (B) Typical ECGs of case 2 showed sinus tachycardia on the left side and III°AVB and elevation of ST segment in I and aVL on the right side. [file Image_2.TIF]

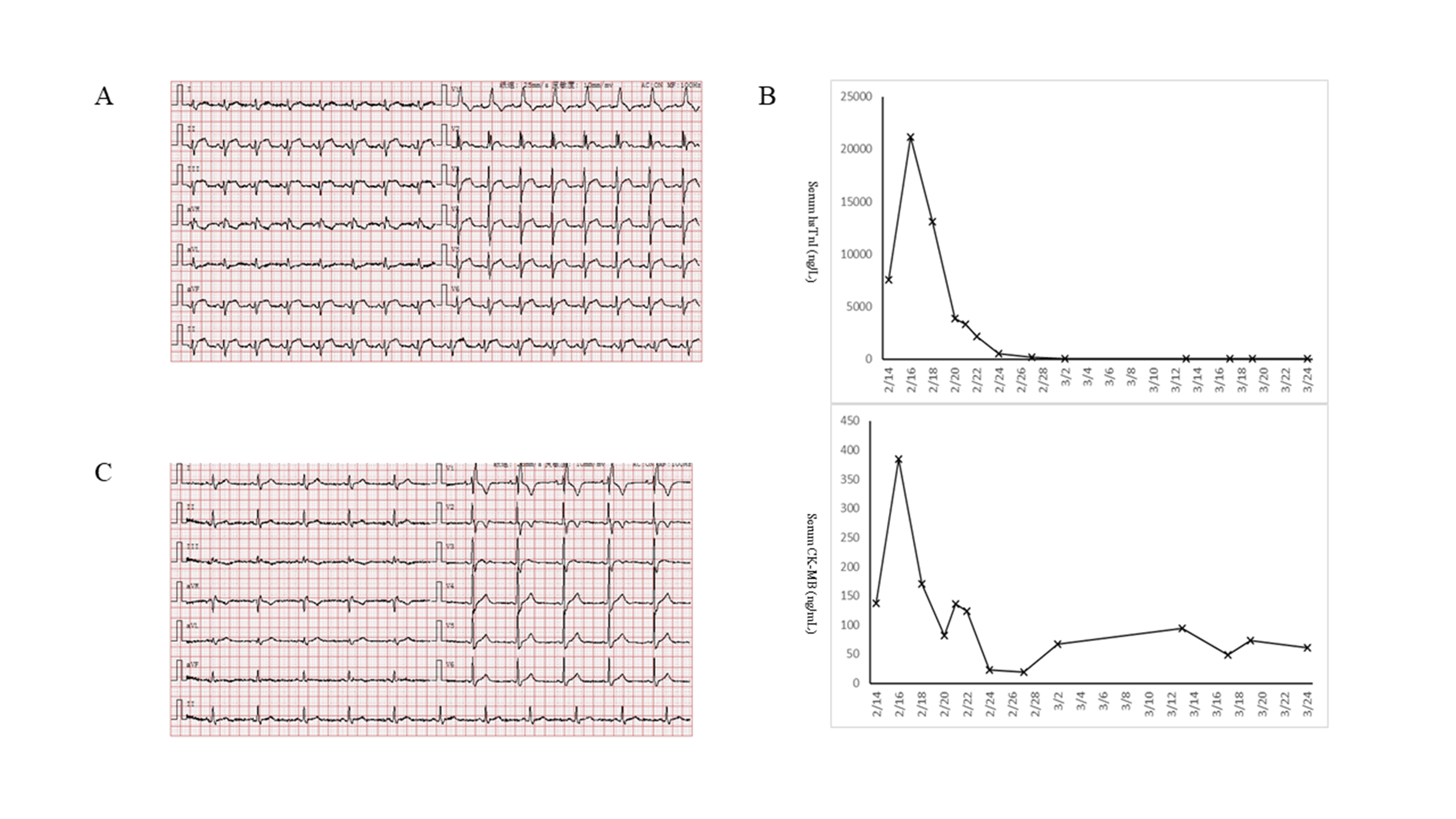

Supplement: Supplementary Figure 3 — Clinical characteristics of case 3. (A) Typical electrocardiogram of case 3 showed elevated ST segment in II, III, aVF and V1-6 leads and complete right bundle branch block. (B) Serum level of hsTnI and CK-MB. (C) Typical electrocardiogram of case 3 showed complete right bundle branch block. [file Image_3.TIF]
