# Supplementary material for: Case Report: Cardiac Toxicity Associated With Immune Checkpoint Inhibitors
Source: Front Cardiovasc Med. 2021 Dec 6;8:727445. doi: 10.3389/fcvm.2021.727445 (PMC8685438; doi:10.3389/fcvm.2021.727445)
Supplement: Supplementary file 5 [file Data_Sheet_1.doc]

**Case Series**

**Case 1**

A 61-year-old male with Stage IV lung cancer was hospitalized for progressively worsening palpitation, chest distress and blepharoptosis in May 2018. The patient had a history of hypertension and was a former smoker with a 20-pack-year history. He was diagnosed with lung squamous cell carcinoma (cT4N2M1) in September 2017 and noted to have disease progression（liver metastasis）after 3 cycles of gemcitabine and cisplatin . Then he received one dose of sintilimab (Anti-PD-1) 200mg intravenously.

Twenty days after sintilimab treatment (3 days before admission) the patient presented with progressively worsening palpitation and accompanying blepharoptosis. On admission the patient showed stable vital signs of blood pressure (BP) 110/60mmHg, palpitation (P) 62 bpm, respiration (R) 18/minute without fever. Electrocardiogram (ECG) indicated atrioventricular block (AVB) progressed from Ⅰ^。^to Ⅲ^。^(supplemental figure 1A) within one day. Echocardiogram revealed acute left ventricular dilation with expanded diameter from 4.3cm to 5.1cm, and acute systolic dysfunction showing ejection fraction (EF) decreased from 56% to 44% within 20 days by M-mode echocardiography. The patient underwent myocardial perfusion imaging and it revealed extensive myocardial perfusion reduction (supplemental figure 1B). Coronary angiography showed no evidence of coronary artery obstruction. The serum level of hypersensitive troponin I (hs-TnI), creatine kinase-MB (CK-MB) and creatine kinase (CK) peaked at 19684.5ng/ml (normal range, 0-26.2ng/ml) ,232.3ng/ml (normal range, 0-6.6ng/ml) and 9714U/L (normal range, 38-174U/L) respectively (supplemental figure 1C). Serum cardiac auto-antibodies against ADP/ATP carrier, β-1-receptor, calcium channel, and myosin were all positive, while serological testing for influenza, coxsackie virus, cytomegalovirus and adenoviruses was found to be negative. Additional findings included leukocytosis and elevated sensitivity C-reactive protein (CRP), glutamic-pyruvic transaminase (ALT) and alanine aminotransferase (AST).

Treatment was given immediately on admission. Methylprednisolone was initiated intravenously at 2mg/kg/day for 5 days, followed by 1.5mg/kg/day for 5 days, and then 10mg/day decrement every 5 days. Additionally, heart failure symptoms were managed with perindopril (4mg per os qd), metoprolol (47.5mg per os qd) and trimetazidine (35mg per os bid). A temporary pacemaker was applied one day after admission when Ⅲ^。^AVB was observed and two weeks later a permanent pacemaker was implanted due to unrecovered Ⅲ^。^AVB. Endomyocardial biopsy was performed one week after methylprednisolone therapy and showed no lymphocytic inﬁltration but only cardiomyocyte degeneration and mild effusion (supplemental figure 1D). One month later, the patient relieved with recovered LV size, cardiac function and cardiac injury markers except blepharoptosis.

Case 2

A 56-year-old woman was referred to our department for fever and chest tightness with a history of thymectomy for B1/B2 thymoma in 2017 and then chemotherapy (gemcitabine and cisplatin) combined with bevacizumab for 5 cycles followed by apatinib, gemcitabine and tislelizumab (anti-PD-1) for 1 cycle because of disease progressed in May 2020. One days after the first administration of tislelizumab, the patient presented with fever and chest tightness. And the serum hsTnI (peak value, 106701.5ng/ml) and CK-MB (peak value, 518.4ng/ml) increased continuously during the course of the disease (supplemental figure 2A). Elevated CK (peak value, 4698 U/L) and BNP (peak value, 725pg/ml) were also noted. Proportion of CD3+ T lymphocyte (92.69%) and CD4+ T lymphocyte (67.91%) were elevated in peripheral blood while NK cells (2.41%) were decreased. Serum IL-6 (93.36pg/ml) and IL-10 (49.01pg/ml) were also in high level. ECG showed progressive cardiac conduction defects from sinus tachycardia to III°AVB, and elevation of ST segment in I and aVL were noted (supplemental figure 2B) within 3 days.

9 days after onset, the patient was diagnosed cardiac irAEs and initiated high dose of methylprednisolone (2mg/kg/d) intravenously as well as trimetazidine and Coenzyme Q10 orally. Aspirin, clopidogrel and statin were also used because myocardial infarction could not be ruled out. And on the 11^th^ day after onset she was admitted to ICU with vital signs (T 38.6℃, BP 126/84mmHg, P 102bpm, R 33/min, SaO2 99% with 3L/min oxygen inhaler). Her arterial blood gas analysis indicated hypoxemia with oxygenation index 375mmHg. The patient was then received plasma exchange twice during the stay of hospital. Despite the treatment, the patient’s serum level of hsTnI kept increasing from 229ng/L to 20595.4ng/L and blood pressure gradually dropped from 120/76mmHg to 84/45mmH within 2 days. There was little clinical improvement and on 14^th^ day after onset, the patient presented sudden cardiac arrest and had no response to cardiopulmonary resuscitation (CPR). As per the family’s wishes, patient was terminally extubated and the further resuscitate attempts were given up.

Case 3

In February 2021, a 52-year-old male patient with thymoma was admitted to our department for blepharoptosis, diplopia, fatigue, chest distress and dyspnea 11 days after one dose of camrelizumab (anti-PD-1) 200mg intravenously. On admission, his vital signs were recorded (BP 142/90mmHg, P85 bpm, T36.5℃, R20/min SaO2 99% in 1L/min oxgen inhaler). Elevated serum hsTnI (peak value, 21180.5ng/L) and CK-MB (peak value, 384.1ng/ml) were observed in this patient (supplemental figure 3A). Echocardiogram revealed wall motion abnormalities in basal segment of the left ventricle. ECG showed elevated ST segment in II, III, aVF and V1-6 leads which lasted for about 3 days and complete right bundle branch block (supplemental figure 3B). Treatments were given 8 days after the onset. The patient received intravenous gamma globulin (0.4g/kg) for 5 days followed by methylprednisolone (500mg/d for 3 days, 240mg/d for 4 days, 120mg/d for 5 days, 60mg/d for 10 days and then 40mg for maintenance dose). Bromostigmine (120mg, qid) and Tacrolimus (0.5mg bid) were also used to alleviated myasthenia. However 10 days after the onset, the patient developed severe respiratory distress. Blood gas analysis revealed acidosis (PH 7.323) and carbon dioxide retention (PaCO2 60.8mmHg, PaO2 126mmHg) when supported by nasal catheter oxygen inhalation (3L/min). Afterwards, the patient received endotracheal intubation and was supported by invasive ventilation for about one week. Due to weaning failure, the patient received tracheotomy and was supported by ventilator for another 10 days. To improve myasthenia, plasma exchange was performed twice. One month after the onset, all the symptoms relieved and hsTnI was reduced to normal level. Electrocardiogram showed consistent complete RBBB (supplemental figure 3C).

Case 4

An 85-year-old male with a history of hepatocellular carcinoma, simultaneous cholecystectomy and hepatectomy was admitted to the hospital with two weeks of progressive dyspnea and disturbance of consciousness in June 2021. He underwent hepatic arterial embolization for recurrent hepatocellular carcinoma 4 months ago and sintilimab (anti-PD1) 200mg for 2 cycles. He developed myasthenia of eyelid and limb and dyspnea one week after the second dose of sintilimab. And the fatigue and dyspnea worsened over the next week, with a progressing manifesting disturbance of consciousness. On admission, his blood pressure was 90/51 mmHg and respiratory rate was 33 breaths/min with an oxygen saturation of 91%. Arterial blood gas analysis indicated hypoxemia combined with carbon dioxide retention (PaO2=50mmHg; PaCO2=70 mmHg). Serum tests showed elevated hsTnI, CK, CK-MB level peaked at 1503.6pg/ml, 10458 U/L, 606.9U/L respectively and elevated LDH at 1186.7U/L. Serum cardiac auto-antibodies against ADP/ATP carrier, β-1-receptor, calcium channel, and myosin were all positive. New onset I°AVB and left bundle branch block (LBBB) with sinus tachycardia (120 beats/min) was indicated, when comparing the present ECG with the previous record. Echocardiogram revealed enlarged left ventricle and acute systolic dysfunction (ejection fraction=40%). CTA only showed 15% stenosis of the circumflex branch of the coronary artery. On admission methylprednisolone was started, and the initial dose was 1000mg/d for 1 days, followed by 500mg/d for 2 days. Meanwhile intravenous gamma globulin was performed at a dose of 0.4g/kg for 2 days. Although the patient suffered from respiratory failure, heart failure and deep coma, endotracheal intubation and mechanical ventilation and were not taken according to the patient's will. The patient developed aggravated carbon dioxide retention, hypoxemia and consciousness disorder even supported by non-invasive mechanical ventilation. 3 days after admission the patient presented sudden cardiac arrest and attempts to resuscitate were given up according to the family's will.

**Case 5**

A 72-year-old male was investigated due to the fortuitous discovery with increased serum hsTnI, CK and CK-MB which peaked at 130.3ng/ml,1298U/L and 18.8ng/mL respectively after ICI therapy although the patient is asymptomatic in June 2019. The patient had a history of right upper lobectomy and lymph node dissection, diagnosed of pulmonary adenocarcinoma (pT2N1M1, stage IV) in January 2019. He was noted to have disease progression after receiving 4 cycles of pemetrexed disodium and carboplatin and 2 cycles of maintenance of docetaxel. Meanwhile radiotherapy was performed on metastatic tumor involving costa, pancreas, lumbar vertebra and local mass. Then he received pembrolizumab（anti-PD-1 antibody）200mg intravenously every three weeks. 20 days after the third pembrolizumab, it was observed that his ECG showed transient nodal tachycardia. And echocardiography revealed mild left ventricular diastolic dysfunction (E/A<1). His electronic medical history was tracked and found that the coronary computer tomography angiography (CTA) 8 month before revealed only mild atherosclerosis for the patient. Trimetazidine (35mg per os bid), coenzyme Q10 (10mg per os tid) and vitamin C (200mg per os tid) were administered. Within one week, his serum hsTnI, CK and CK-MB decreased to normal level, and his ECG was normal while mild increased heart rate (95 bpm) still remain. Three weeks after his recovery, the patient rechallenged the pembrolizumab therapy and showed no signs of cardiac injury during the following 6 cycles of ICI therapy.

**Case 6**

A 58-year-old female patient with right lower lobectomy and postoperative diagnosis IIIB stage of lung adenocarcinoma was admitted to our department for palpitation and chest tightness in July 2019. The patient had a history of papillary thyroid carcinoma in complete remission since thyroidectomy 5 years ago. After 4 cycles of docetaxel and nedaplatin lung cancer progression was noted. Then she received 3 cycles of pemetrexed and carboplatin, combined with pembrolizumab(anti-PD-1). 22 days after her third cycle of pembrolizumab(200mg) intravenously, the patient presented with palpitation and chest tightness. At onset, she showed stable vital signs (BP 132/72mmHg, P 104bpm, R 20bpm, T 36.5℃). Laboratory tests showed elevated hs-TnI level peaked at 1000.6pg/ml. ECG showed transient nodal tachycardia and echocardiogram revealed mild left ventricular diastolic dysfunction (E/A<1). Coronary artery computed tomography angiography (CTA) observed no abnormality. 16 days after the onset, methylprednisolone (40mg/d for 7 days, 30mg/d for 7 days, 20mg/d for 7 days and 10mg/d for 7 days) as well as trimetazidine (35mg bid) and metoprolol (47.5mg qd) were administered. Although elevated hsTnI lasted for 6 weeks, the patient’s symptoms relieved two days after the treatment immediately. No unusual change in ECG was noted since onset. Four months later the patient restart pemetrexed chemotherapy but ICI was stopped.

**Case 7**

A 45-year-old female patient was diagnosed with breast cancer in 2011 and cervical cancer (IIa) in 2015. She initiated her chemotherapy since 2015 and was treated with irinotecan and nedaplatin for 2 cycles, cisplatin for 4 cycles, GP schemes (gemcitabine and nedaplatin) and Bevacizumab for 2 cycle, and then TP schemes for 4 cycles. She also received synchronous radiotherapy for 4 times. Then she was enrolled in a phase II clinical trial through which she received ZKAB001, a PD-L1 monoclonal antibody every two weeks at the dose of 5mg/kg in June 2019. Ten days after her second usage of ZKAB001, the patient presented right blepharoptosis and admitted to our hospital. Serum hs-TnI levels elevated moderately and peaked at 100ng/ml. ECG showed nodal tachycardia and echocardiogram showed mild left ventricular diastolic dysfunction (E/A<1) as well as little pericardial effusion. Cardiac magnetic resonance imaging (CMR) was normal. One day after onset, methylprednisolone (100mg/d for 3 days, 80mg/d for 3 days, 60mg/d for 9 days, 40mg/d for 3 days, and 20mg/d for 2 days) as well as trimetazidine (35mg bid), Coenzyme Q10 and metoprolol (47.5mg qd) were performed. The level of hsTnI decreased to normal two weeks after the treatment. And blepharoptosis relieved 3 weeks after treatment. Four months later the patient restart pemetrexed therapy with ICI discontinuation.

Case 8

A 77-year-old male patient with a prior history of coronary stenting and pyriformsinus squamous cell carcinoma was admitted to our hospital for tumor maintenance therapy in May 2021. He underwent complete laryngectomy and receive local radiation therapy twice in 2019. Afterwards pulmonary metastasis was noted on CT scan and then the patient received TP (paclitaxel and cisplatin) schemes for chemotherapy and sintilimab (anti-PD1) 200mg twice. 3 days after his second usage of sintilimab, elevated hsTnI (peak value 2615.2ng/L) was noted in routine monitoring while serum CK and CK-MB were in normal level. However the patient was asymptomatic with stable vital signs (BP 115/85mmHg, P 96bpm, T 36.3℃, R 20/min). Electrocardiogram showed sinus tachycardia. Echocardiogram observed mild ventricular diastolic dysfunction (E/A<1). Treatments were given immediately when elevated hsTnI was noted. Methylprednisolone (40mg qd) was used for 3 days, and was then added to 100mg qd due to increasing hsTnI. Methylprednisolone (100mg/d) was used for another 3 days and the level of hsTnI decreased. Then methylprednisolone was reduced by 20mg every 3 days. Two weeks later his serum level of hsTnI decreased to normal line. The patient restarted his chemotherapy one month later but stopped ICI treatment.

Case 9

A 70-year-old female patient with history of hypertension and gallbladder carcinoma accompanied by liver metastases was admitted to our hospital for tumor maintenance therapy in April 2021. The patient received radical resection in 2019 and then experienced gemcitabine combined with tegafur, gimeracil and oteracil porassium capsules for 7 cycles in the successive 7 months. She was noted to have cancer progression after, CT scan revealed enlarged Virchow node in June 2020. Afterwards, the patient received local radiation therapy, paclitaxel and capecitabine for 5 cycles, followed by combined with camrelizumab (anti-PD1) 200mg every three weeks. 10 days after the 5^th^ use of camrelizumab, elevated CK-MB (7.2ng/ml) and CK (480U/L) were noted whereas hsTnI was negative. Since the patient was asymptomatic, she chose clinical observation and did not receive any therapy. After 15 days, serum CK-MB peaked at 10.5ng/ml while CK peaked at 725U/L. ECG revealed I°AVB. Maybe because the detection time window was missed, her serum hsTnI stayed normal. However based on the appearance of AVB, methylprednisolone (32mg qd) with a decrement of 8mg every week was administered to avoid further myocardial injury. After being treated for one week, her serum CK-MB was reduced to normal and ECG showed sinus arrhythmia.

Case 10

A 64-year-old male with IIIA stage of lung squamous cell carcinoma (cT2N2M0) was admitted to the department for palpitation and blepharoptosis in August, 2021. He was diagnosed with lung cancer and initiated the albumin paclitaxel and cisplatin combined with camrelizumab (anti-PD1) in July 2021. 15 days after the second cycle of camrelizumab (200mg), the patient presented with progressive palpitation and blepharoptosis. At onset, he showed racing heart at 120bpm, rapid breath at 22bpm, BP 132/72mmHg and T 36.5℃. Laboratory tests showed elevated hs-TnI, AST and CK level peaked at 516pg/ml, 425U/L and 6126U/L respectively. Additional findings included elevated CRP (8.68mg/L) and ALT (peaked at 201U/L). ECG showed transient nodal tachycardia and echocardiogram revealed mild left ventricular diastolic dysfunction (E/A<1). 7 days after the onset, methylprednisolone (40mg/d for 7 days, 30mg/d for 7 days, 20mg/d for 7 days) as well as trimetazidine (35mg bid) and metoprolol (47.5mg qd) were performed. After being treated for one week, her serum hs-TnI was reduced to normal and ECG showed sinus arrhythmia. The patient was performed ICI discontinuation.
